# Effects of drug concentration and PLGA addition on the properties of electrospun ampicillin trihydrate-loaded PLA nanofibers

**DOI:** 10.3762/bjnano.13.19

**Published:** 2022-02-21

**Authors:** Tuğba Eren Böncü, Nurten Ozdemir

**Affiliations:** 1 Faculty of Pharmacy, Department of Pharmaceutical Technology, Erciyes University, 38280 Kayseri, Turkey; 2 Faculty of Pharmacy, Department of Pharmaceutical Technology, Ankara University, 06560 Ankara, Turkey

**Keywords:** ampicillin trihydrate, electrospinning, nanofiber, PLA nanofiber, PLA/PLGA nanofiber

## Abstract

The aim of this study was to produce ampicillin trihydrate-loaded poly(lactic acid) (PLA) and PLA/poly(lactic-*co*-glycolic acid) (PLA/PLGA) polymeric nanofibers via electrospinning using 1,1,1,3,3,3-hexafluoro-2-propanol (HFIP) as the solvent for local application in tissue engineering. The effects of ampicillin trihydrate concentration (4–12%) and addition of PLGA (20–80%) on the spinnability of the solutions, morphology, average nanofiber diameter, encapsulation efficiency, drug release, and mechanical properties of PLA and PLA/PLGA nanofibers were examined. All nanofibers were bead-free and uniform. They had favorable encapsulation efficiency (approx. 90%) and mechanical properties. The increase in the amount of ampicillin trihydrate caused an increase in the diameter and burst effect of the nanofibers. The drug release ended on the 7th and 3rd day with nanofibers containing 4% and 12% of drug, respectively. The prolonged and controlled drug release for ten days was obtained with nanofibers containing 8% of drug. Thus, the ideal drug concentration was determined to be 8%. Nanofibers containing PLA/PLGA had a larger diameter than those including PLA. In addition, both the strength and elongation of nanofibers decreased depending on the increase in nanofiber size with the addition of PLGA, increased amount of drug, and ratios of PLGA. Drug release studies showed that PLA/PLGA nanofibers exhibited a lower burst effect and a decrease in drug release when compared to PLA nanofibers. Finally, PLA/PLGA nanofibers can be produced with enhanced encapsulation efficiency and mechanical properties, resulting in controlled and tailored release of ampicillin trihydrate for at least ten days. In conclusion, it was demonstrated that the addition of PLGA in different ratios and the amount of drug can be manipulated to obtain the desired properties (average nanofiber diameter, morphology, in vitro drug release, and mechanical properties) of PLA nanofibers.

## Introduction

Polymeric nanofibers have been widely used in many fields such as tissue engineering and drug delivery systems. Electrospinning is the most commonly used polymeric nanofiber preparation method, because it is an easy, single-step, low-cost, and reproducible method. It allows for the production of extracellular matrix-like nanofibers that can be easily scaled up and has different properties with many polymers and solvents [1–4]. Drug-loaded electrospun polymeric nanofibers have many unique properties, such as accelerating healing, controlled drug release, stimulation of cell growth and proliferation due to their similarity to the extracellular matrix, large surface area, high encapsulation efficiency, high porosity, and superior mechanical properties [5–7].

In our study, FDA-approved polylactic acid (PLA) and poly(lactic-*co*-glycolic acid) (PLGA), which are frequently preferred polymers in the production of polymeric nanofibers, were used because they are biodegradable, biocompatible, non-toxic, and provide high mechanical strength [1,8]. In this study, ampicillin trihydrate, FDA-approved β-lactam antibiotics, a broad-spectrum semi-synthetic derivative of aminopenicillin, was used. Ampicillin trihydrate acts by inhibiting the synthesis of peptidoglycan, a critical component of the bacterial cell walls [9]. 1,1,1,3,3,3-Hexafluoro-2-propanol (HFIP) was used as solvent in the study. It is preferred due to its sufficiently low surface tension, sufficiently high dielectric constant, and volatility [10].

The aim of this study was to produce and characterize ampicillin trihydrate-loaded implantable PLA and PLA/PLGA polymeric nanofibers for controlled drug release with favorable properties for the use in tissue engineering. In this study, ampicillin trihydrate-loaded PLA and PLA/PLGA nanofibers with acceptable morphology, nanofiber diameter, mechanical properties, encapsulation efficiency, and controlled drug release were prepared via electrospinning. The spinnability and properties of PLA nanofibers associated with drug concentration (4–12%) and PLGA addition (20–80%) were also investigated. A limited number of studies examining electrospun PLA/PLGA nanofibers have focused on drug-free or hydrophilic drug-loaded PLA/PLGA nanofibers produced with different polymers and solvent systems than those in the current study [8,11]. The current study is important since the effects of PLA/PLGA ratios on nanofiber morphology, nanofiber diameter, in vitro drug release, and mechanical properties are examined. The study will also contribute to the production of implantable systems of ampicillin trihydrate, a hydrophobic antibiotic, with a controlled release. Thus, it will allow improvement in treatment efficiency with lower doses of antibiotics, reduce systemic side effects, prevent antibiotic resistance, and increase patient compliance. In our previous study, ampicillin trihydrate-loaded electrospun PLA and PLA/PCL nanofibers were produced and the effect of PLA concentration, addition and amount added of PCL on the nanofibers properties were investigated [12]. These studies will make fundamental contributions to the investigation of electrospun PLA and composite (PLA/PLGA and PLA/PCL) nanofibers.

## Results and Discussion

### Preparation and characterization of ampicillin trihydrate-loaded electrospun nanofibers

PLA and PLA/PLGA nanofibers prepared in this study, plus the average nanofiber diameters calculated in ImageJ using scanning electron microscopy (SEM) images of nanofibers, are given in Table 1 and Table 2.

**Table 1:** PLA nanofibers prepared in the study.

Formulation	F1	F2	F3

polymer	PLA	PLA	PLA
polymer concentration (%)	10	10	10
polymer ratio (%)	100:0	100:0	100:0
ampicillin trihydrate (%)	4	8	12
voltage (kV)	11.5	11.5	11.5
capillary–collector distance (cm)	10	10	10
flow rate (mL/h)	0.8	0.8	0.8
diameters ± SD (nm)	416.5 ± 8.4	432.7 ± 11.4	476.7 ± 9.8
encapsulation efficiency (%)	91.3	90.0	64.5

**Table 2:** PLA/PLGA nanofibers prepared in the study.

Formulation	F2	F4	F5	F6

polymer	PLA	PLA/PLGA	PLA/PLGA	PLA/PLGA
polymer concentration (%)	10	10	10	10
polymer ratio (%)	100:0	80:20	60:40	20:80
ampicillin trihydrate (%)	8	8	8	8
voltage (kV)	11.5	11.5	11.5	11.5
capillary–collector distance (cm)	10	10	10	10
flow rate (mL/h)	0.8	0.8	0.8	0.8
diameters ± SD (nm)	432.7 ± 11.4	820.0 ± 10.4	747.9 ± 14.7	447.1 ± 6.6
encapsulation efficiency (%)	90.0	89.4	89.9	91.2

**Figure 1:**
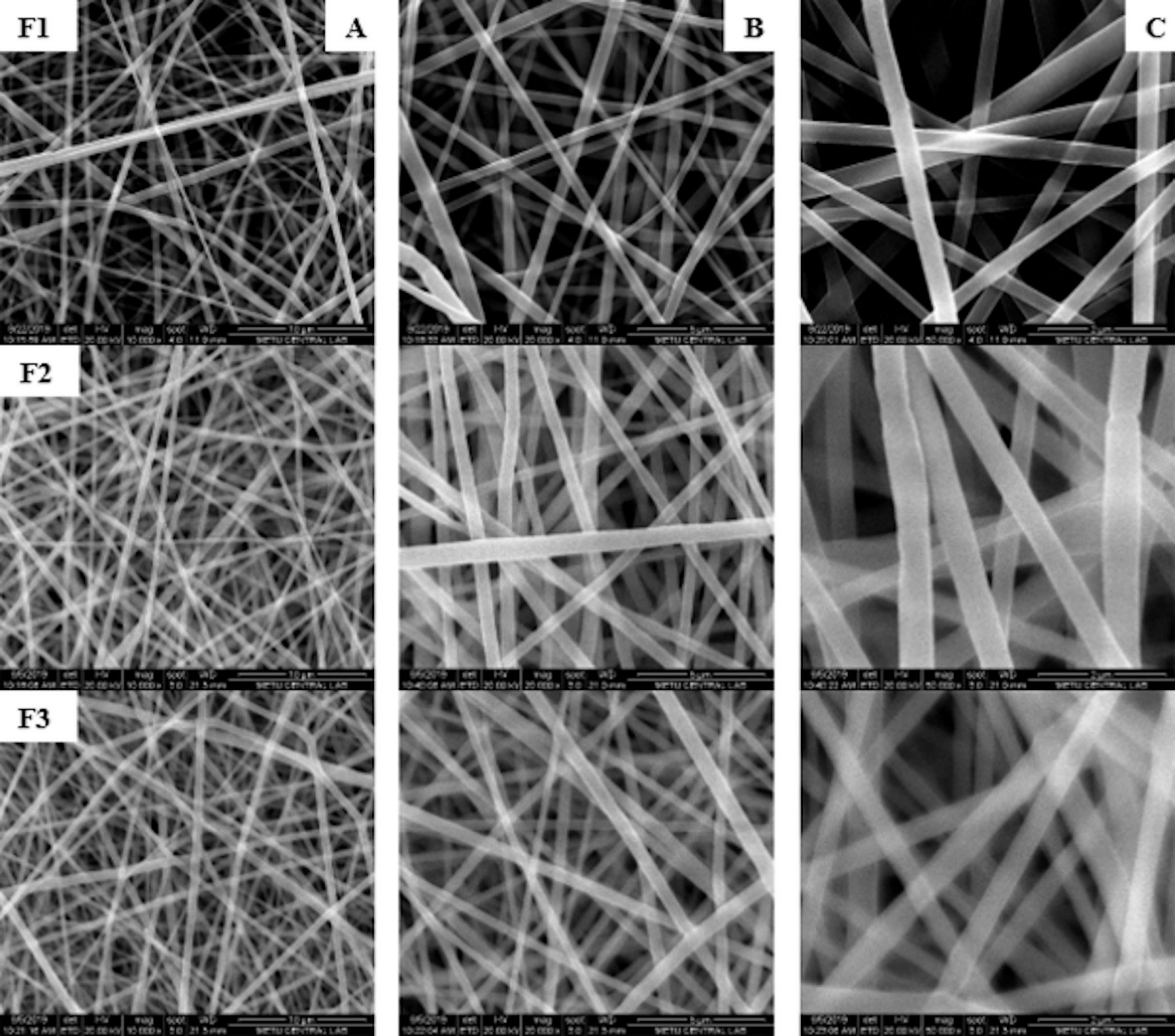
SEM images of nanofibers produced by changing the ampicillin trihydrate concentration (F1: 4%, F2: 8%, and F3: 12%) (A: 10.000×, B: 20.000×, C: 50.000×).

**Figure 2:**
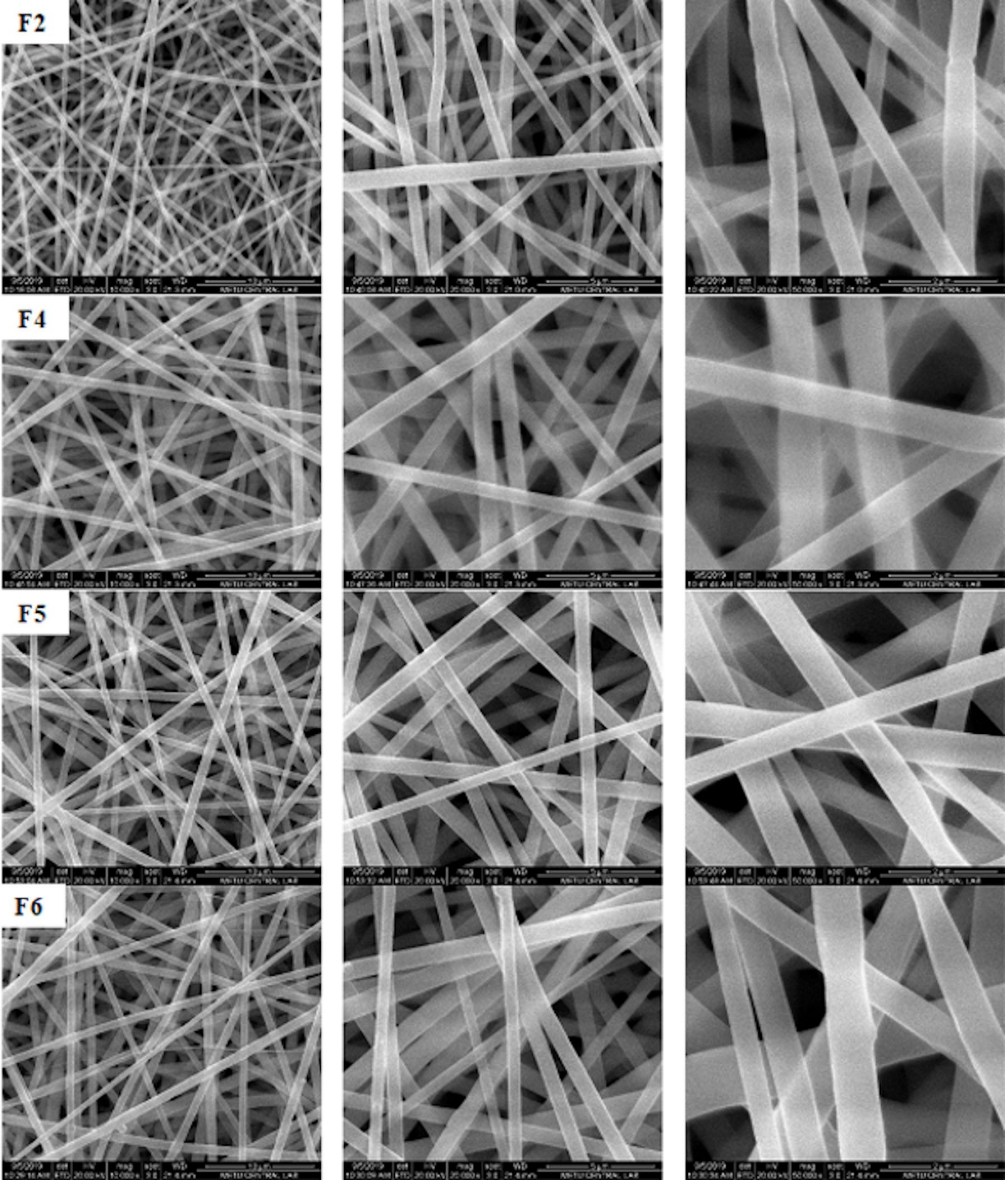
SEM images of nanofibers produced by different ratios of PLA/PLGA [F2: PLA (100:0); F4: PLA/PLGA (80:20); F5: PLA/PLGA (60:40); F6: PLA/PLGA (20:80)].

Stable jet and continuous nanofiber formation was observed in all PLA nanofibers containing different amounts of drug and in PLA/PLGA nanofibers with different ratios of PLGA. All PLA and PLA/PLGA nanofibers showed randomly aligned, smooth, and bead-free morphology (Figure 1 and Figure 2).

The diameters of the PLA nanofibers ranged from 417 to 477 nm. While the diameter of the nanofiber containing 4% of drug was 417 nm, the diameter of the nanofiber increased to 433 and 477 nm when the amount of drug was increased to 8% and 12%, respectively (*p* < 0.05). As the amount of drug in the nanofiber increased, the nanofiber diameter also increased. This could be attributed to the increase in the amount of drug, resulting in surface loading [13].

In order to examine the effects of PLGA addition on PLA nanofibers, PLA/PLGA nanofibers were produced by replacing 20 to 80% of PLA with PLGA in the F2 coded formulation containing 8% of drug. As seen in Table 2, the diameters of the PLA/PLGA nanofibers range from 447 to 820 nm. The addition of PLGA to PLA led to an increase in nanofiber diameter (*p* < 0.05). The diameters of the nanofibers showed an increase from 433 to 820 nm with the addition of 20% of PLGA in comparison to nanofibers containing only PLA. However, upon an increase in the percentage of PLGA added (from 40 to 80%), nanofiber diameters were found to be 748 and 447 nm, respectively. These obtained values did not meet the expected nanofiber diameter increase that would have been achieved in correlation with the PLGA percentage increase. The increase in nanofiber diameter with the addition of PLGA can be explained by the higher molecular weight of PLGA than that of PLA since the increase in polymer molecular weight increases the viscosity, causing an increase in nanofiber diameter [14–16]. In other studies conducted on PLA/PLGA nanofibers, it was found that an increase in the viscosity of the solutions caused an increase in nanofiber diameters [8]. It was also claimed that the viscosity, which is related to the molecular weight of the polymer and the concentration of the polymer solution, is one of the most effective parameters to tune fiber diameters [11].

The nanofiber diameter is also greatly affected by changes in polymer structure [17–18] and crystallization properties of the polymer [19]. Since the crystallinity of PLGA is known to be lower than that of PLA [20] and its structure is different, the increase in diameter with the addition of PLGA may be related to the lower crystallinity and different structure of PLGA.

In our previous study, we observed that the addition of PCL (20–80%) to PLA nanofibers caused an increase in fiber diameter [12]. In both studies, the addition of different polymers (both PLGA and PCL) to PLA nanofibers resulted in an increase in fiber diameter. In addition, in both studies, the diameter of the largest fiber was produced by adding 20–40% of polymer to PLA, and there was less increase in diameter by adding 80% of polymer. All these data show that the addition of different polymers causes an increase in the diameter of PLA nanofibers. However, this increase is not always linear with the amount of polymer added.

Hiep et al. found that an increase in the amount of PCL in PLGA/PCL nanofibers from 10% to 20% causes a decrease in the fiber diameter from 1000 to 500 nm, while an increase in the amount of PCL to 30% causes an increase in the diameter to 2000 nm [1]. This study also supports the conclusion that the addition of a different polymer to the electrospinning solution causes a change in nanofiber diameters independently of the amount of polymer added.

### Encapsulation efficiency of nanofibers

It has been shown that all nanofibers exhibited superior encapsulation characteristics. The encapsulation efficiency of PLA nanofibers containing up to 8% of drug was quite high. A significant decrease in encapsulation efficiency was observed with an increase in the drug content to 12% (Table 1, *p* < 0.05). While the encapsulation efficiency of nanofibers containing 4% and 8% of ampicillin trihydrate was approx. 90%, it decreased to 65% when the amount of ampicillin trihydrate was increased to 12%. It is thought that the encapsulation efficiency is reduced due to of the undissolved drug in the solution [21]. In addition, F3 coded nanofiber containing 12% of ampicillin trihydrate may have formed a heterogeneous matrix instead of a homogeneous one.

The addition of PLGA with different concentrations did not cause any changes in the encapsulation efficiency. All PLA/PLGA nanofibers were shown to have favorable encapsulation efficiency (approx. 90%).

### Dissolution studies

**Figure 3:**
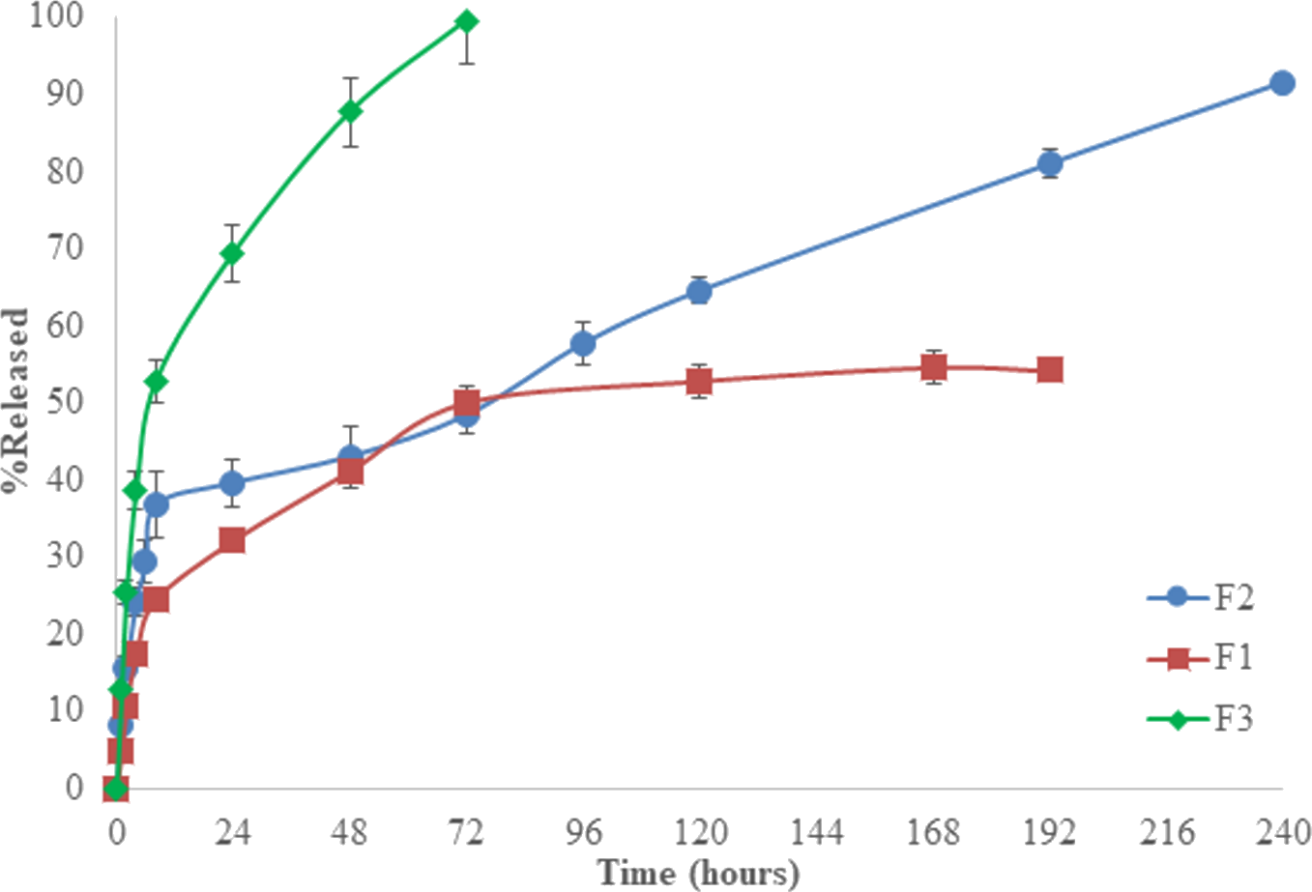
Effect of drug amount (F1: 4%, F2: 8%, and F3: 12%) on drug release from PLA nanofibers.

In vitro drug release from PLA and PLA/PLGA nanofibers was investigated by using the static method. The in vitro drug release of PLA electrospun nanofibers produced by varying the amount of ampicillin trihydrate is shown in Figure 3.

By increasing the amount of drug used in formulations, the burst effect was increased. Cumulative drug release at 24 h was 32.1%, 39.6%, and 69.4% for formulations containing 4% (F1), 8% (F2), and 12% (F3) of ampicillin trihydrate, respectively. While the drug release ended within three days in F3 (containing 12% of drug) and on the 7th day in F1 (containing 4% of drug), drug release was considerably extended up to ten days for the F2 coded formulation (containing 8% if drug) (Figure 3). In other studies conducted on different polymers and polymer blends and drugs, it was shown that the increase in the amount of drug caused a higher burst effect and faster drug release [13,22–23]. It was concluded that the optimum concentration of ampicillin trihydrate in PLA nanofibers was 8% for a controlled drug release.

**Figure 4:**
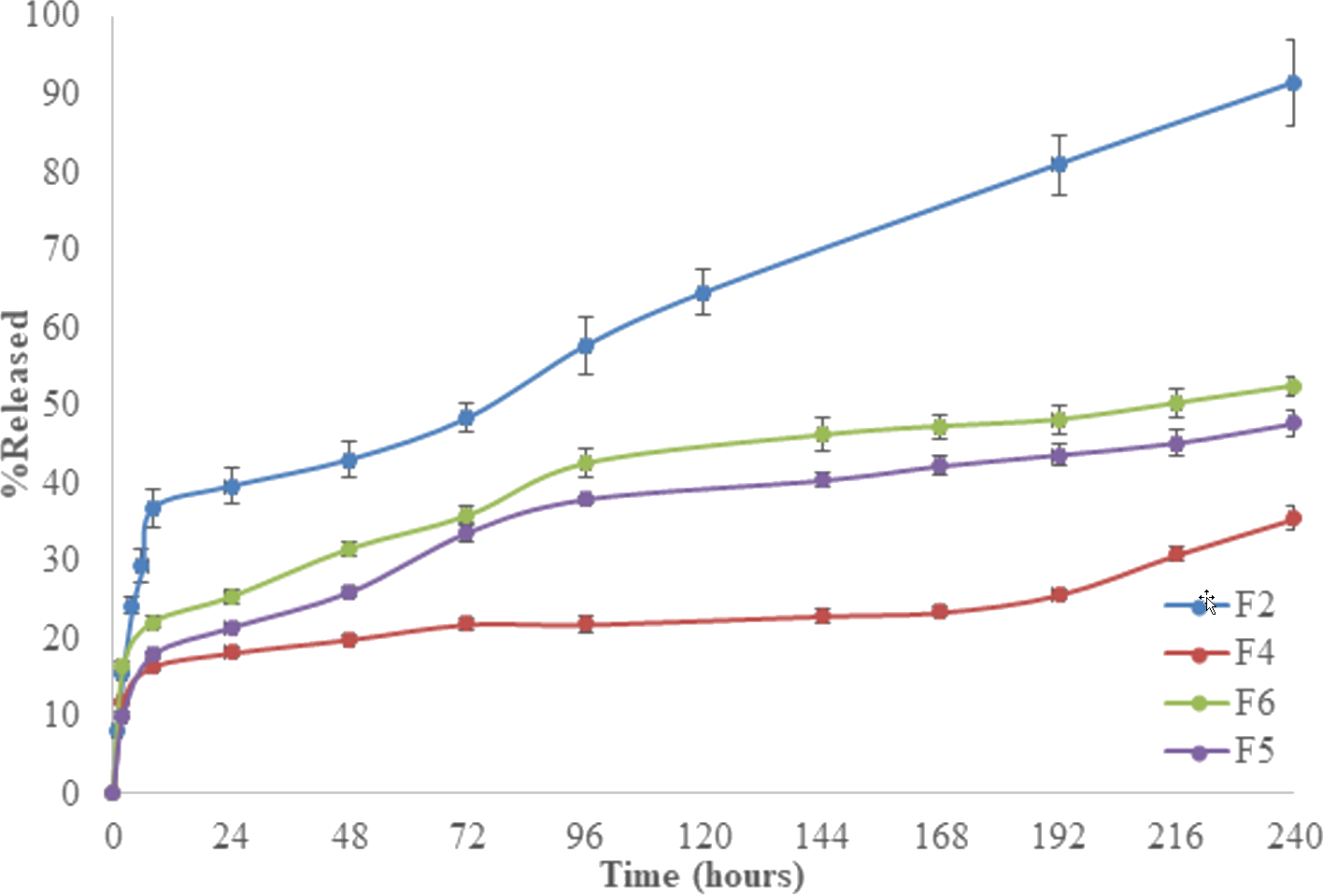
Effect of different ratios of PLA/PLGA on drug release from nanofibers [F2: PLA (100:0); F4: PLA/PLGA (80:20); F5: PLA/PLGA (60:40); F6: PLA/PLGA (20:80)].

In vitro drug release of PLA/PLGA electrospun nanofibers produced by different ratios of PLA/PLGA are shown in Figure 4.

PLA/PLGA nanofibers had a lower burst effect and slower drug release compared to PLA nanofibers. In addition, as the amount of added PLGA was increased, the burst effect and release rate were slowed. As seen in Table 2 and Figure 4, nanofiber diameters are thicker and drug release is slower upon the addition of PLGA. The reasons for the reduction in drug release in the presence of PLGA are: i) The viscosity of PLGA/PLA solutions is higher due to the higher molecular weight of PLGA than that of PLA [14,24], resulting in thicker PLGA/PLA nanofibers. Drug release was slower in nanofibers with a larger diameter (PLGA/PLA) than in nanofibers with a smaller diameter (PLA) due to the greater distance required for the drug to diffuse and lower specific surface areas relative to fibers with a smaller diameter [16,25–26]. ii) The structure of PLGA is different than that of PLA. The type of polymer changes the release properties as it has a significant effect on the intramolecular and intermolecular interactions that affect the physical properties of the electrospinning solution, thus leading to differences in the drug release properties of the electrospun nanofibers [2,24]. iii) PLGA is more hydrophilic compared to PLA [11]. iv) The increase in the molecular weight reduces the nanoporosity of the nanofibers, resulting in a slower drug release [14,24]. Batista et al. also claimed that the composition of the PLA/PLGA blend affected the release of gentamycin sulfate due to the differences in hydrophobicity of PLA and PLGA [11].

In our previous study, we examined the effect of adding another synthetic polymer, PCL, to PLA nanofibers. Higher burst effects were observed and drug release was significantly accelerated by adding increasing amounts of PCL to PLA nanofibers [12]. These results show that the drug release from nanofibers is also affected by the properties of the polymers added to PLA. As a result, the hydrophobic antibiotic ampicillin trihydrate had the lowest burst effect and the slowest controlled release with PLA/PLGA nanofibers compared to that of PLA, PCL and PLA/PCL nanofibers.

**Figure 5:**
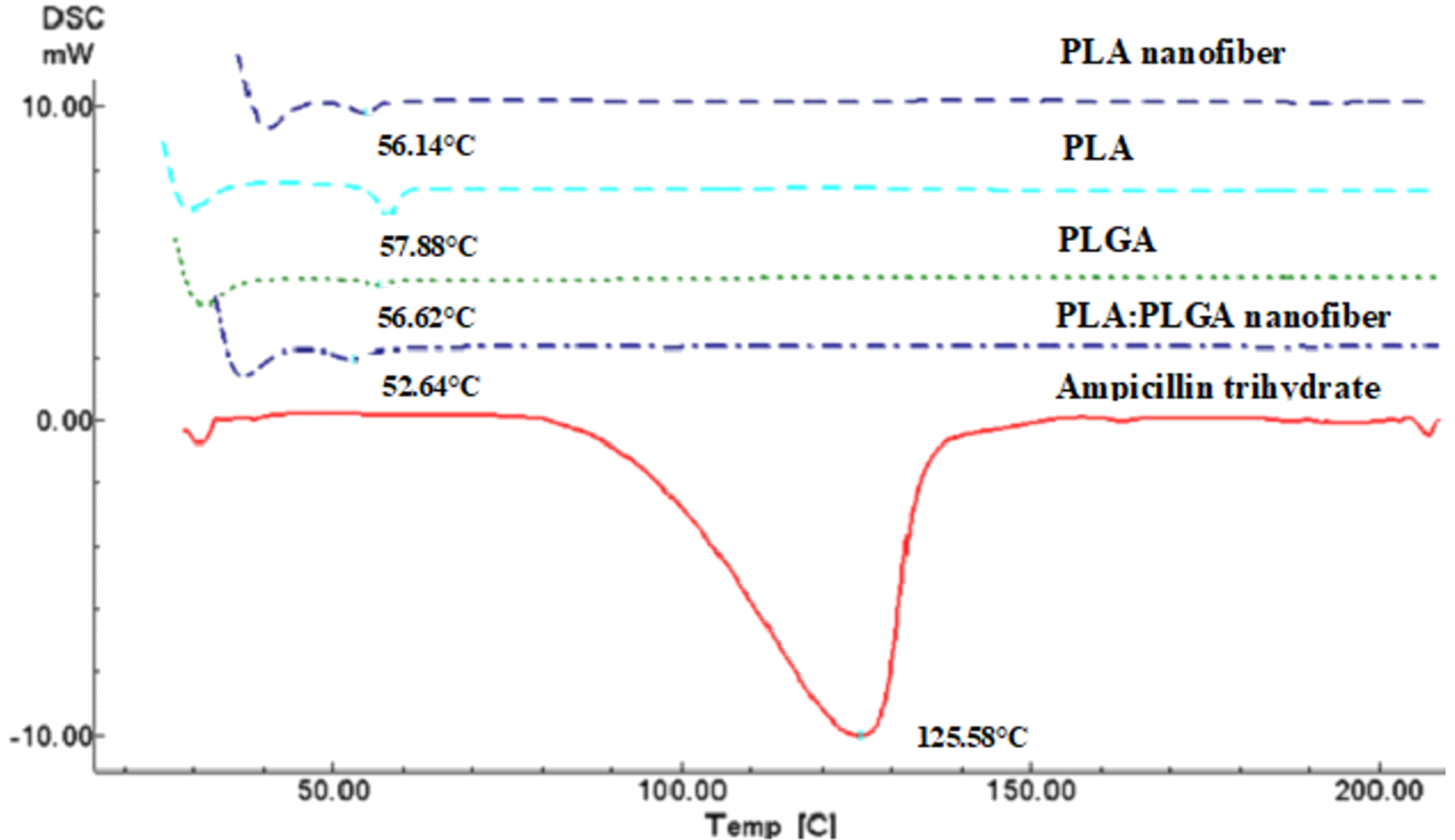
Differential scanning calorimetry analysis (DSC) thermograms of ampicillin trihydrate, PLA, PLGA, PLA nanofibers, and PLA/PLGA nanofibers.

As shown in Figure 5, the absence of the melting endotherm peak at 125.58 °C specific to pure ampicillin trihydrate on the DSC thermograms of PLA, PLGA, PLA nanofibers, and PLA/PLGA nanofibers proves that ampicillin trihydrate was loaded in the nanofibers in an amorphous form.

### Mechanical properties of nanofibers

The mechanical properties of nanofibers depend on their composition, porosity, average size, distribution, individual nanofiber orientation, interaction between nanofibers, and arrangement and entanglement of the nanofibers [27–29].

When the mechanical properties of PLA nanofibers containing different amounts of drug were examined, the increase in the amount of drug caused an increase in nanofiber size (Table 1), resulting in both lower tensile strength and elongation values (*p* < 0.05) (Table 3).

**Table 3:** Mechanical properties of PLA nanofibers.

Formulation	Tensile strength ± SD (mPa)	Elongation at break ± SD (%)

F1	2.62 ± 0.46	21.59 ± 7.51
F2	2.06 ± 0.34	11.64 ± 0.95
F3	1.77 ± 0.24	9.52 ± 1.26

The values of tensile strength and elongation at break of PLA nanofibers containing 4% of drug were 2.62 mPa and 21.59%, respectively. When the amount of drug was increased from 4% to 8%, the aforementioned values decreased to 2.06 mPa and 11.64%, while the nanofiber diameter increased from 417 to 433 nm, respectively (*p* < 0.05). Similarly, the increase in the amount of drug to 12% caused an increase in the nanofiber diameter and a decrease in mechanical properties values (*p* < 0.05). This was similar to our previous studies with linezolid-loaded PLGA and PCL/PLGA nanofibers [16,30]. Chew et al. (2006) also showed that an increase in bovine serum albumin caused an increase in nanofiber diameter and a decrease in mechanical properties of poly(caprolactone-*co*-ethyl ethylene phosphate) nanofibers [31].

The tensile strength value of PLA nanofibers was 2.06 mPa and the elongation at break was 11.64%. When the PLGA concentration was 20% (F4), 40% (F5), and 80% (F6), the tensile strength was 2.58, 2.66 and 2.15 mPa, respectively (Table 4). As PLGA was added to PLA nanofibers, the mechanical property values of PLA/PLGA nanofibers generally increased (except F6) and the nanofibers had a harder structure.

**Table 4:** Mechanical properties of PLA/PLGA nanofibers.

Formulation	Tensile strength ± SD (mPa)	Elongation at break ± SD (%)

F2	2.06 ± 0.34	11.64 ± 0.95
F4	2.58 ± 0.27	12.46 ± 1.04
F5	2.66 ± 0.20	11.49 ± 0.40
F6	2.15 ± 0.17	11.94 ± 0.85

The improvement in the mechanical properties of nanofibers with the addition of PLGA can be explained as follows: i) The nanofiber size affects the deformation behavior as fibers with a larger diameter tend to display bulk-like properties and have a compact arrangement and stable structure [8,32]. ii) An increase in diameter causes a decrease in porosity [8]. iii) PLGA has more enhanced mechanical properties that those of PLA [33]. In addition, Zhang et al. stated that combined use of PLA and PLGA, compared to the use of PLA or PLGA alone, improved the mechanical properties and led to the production of more rigid structures with higher tensile strength [7]. In our previous study, the values of the mechanical properties of PLA/PCL nanofibers were reduced compared to those of PLA nanofibers. This was explained by the fact that PLA has higher mechanical properties compared to those of PCL [12].

As a result, it has been proven that the type of polymer added to PLA nanofibers and the mechanical properties of the polymer have a direct effect on the mechanical properties of nanofibers. Enhanced mechanical properties are known to improve cell viability and differentiation [34]. The mechanical properties of all PLA nanofibers (PLA, PLA/PCL, and PLA/PLGA) are suitable. However, the production of PLA/PLGA nanofibers provides an advantage as it leads to improved mechanical properties compared to those of PLA nanofibers and PLA/PCL nanofibers, improving cell viability and differentiation.

## Conclusion

Nanofibers can be effectively used in tissue engineering and controlled drug delivery due to their structural properties, which are morphologically similar to those of the extracellular matrix. Ampicillin trihydrate-loaded smooth, bead-free electrospun PLA and PLA/PLGA nanofibers have been successfully developed as an implantable system. All nanofibers have favorable encapsulation efficiency and mechanical properties. The increase in the amount of drug (from 4% to 12%) in the PLA nanofiber led to an increase in the nanofiber diameter and resulted in a higher burst effect and faster drug release. F2 coded nanofiber was chosen as the ideal PLA nanofiber with ideal drug concentration (8%) due to its favorable encapsulation efficiency, nanofiber diameter, morphology and mechanical properties, and its ability to allow for the best controlled drug release for at least ten days.

PLA/PLGA nanofibers containing 8% of drug and different proportions of PLGA (20–80%), have a lower burst effect and slower drug release compared to PLA nanofibers. However, as the amount of PLGA used in the production of PLA/PLGA electrospun nanofibers were increased, the initial burst effect and release rate were also increased.

The reasons for the reduction in drug release in the presence of PLGA are: i) PLGA has a higher molecular weight than that of PLA, and PLA/PLGA nanofibers have thicker diameters due to the higher viscosity of PLGA/PLA solutions, ii) the difference in structure and hydrophobicity of PLGA causes differences in the intermolecular interactions of the electrospinning solution.

The increase in the amount of drug caused an increase in the nanofiber diameter and thus a decrease in the mechanical properties of the PLA nanofibers. PLA/PLGA nanofibers may be advantageous for improving cell viability and differentiation thanks to its advanced mechanical properties compared to those of PLA nanofibers. Nevertheless, the mechanical properties of all nanofibers produced in the study are appropriate for the use in tissue engineering.

From our study, it may be concluded that the characteristics of the nanofibers (average nanofiber diameter, mechanical properties, and drug release) can be tailored by manipulating the addition of PLGA, a different type of polymer, the amount of added drug, and PLGA. As a result, it is advantageous to produce PLA and PLA/PLGA nanofiber mats via electrospinning, with favorable encapsulation efficiency (approx. 90%) and mechanical properties and a tailored and controlled release for approx. ten days for the use in tissue engineering. It has also been concluded that local application of drug-loaded nanofibers could reduce the systemic side effects caused by the drug and increase patient compliance and treatment efficacy.

## Experimental

### Materials

Ampicillin trihydrate was obtained from Atabay (Istanbul, Turkey) as a gift. PLA (MW of 103000 g/mol), ester-terminated PLGA (MW of 190000–240000 g/mol, a lactide/glycolide ratio of 85:15), and HFIP were obtained from Sigma-Aldrich. All the other chemicals used were of analytical grade.

### Electrospinning

Nanofibers were produced via the electrospinning method in a similar manner as described by our previous study [16]. PLA nanofibers and PLA/PLGA nanofibers prepared in the study are given in Table 1 and Table 2, respectively. A polymer solution (10%, w/v) was prepared by dissolving the polymer in HFIP. Then, 4–12% w/w of ampicillin, based on the dry weight of polymer, was dissolved in the polymer solutions. For electrospinning, the solutions were poured into a plastic syringe (5 mL) fitted with a 21 G needle. The syringe was then placed in a syringe pump and a high voltage was applied between the needle and the grounded stationary rectangular metal collector. The process parameters used in the current study are shown in Table 1 and Table 2 (Electrospinning machine Ne-200, Inovenso, Turkey). The collector covered by a piece of aluminum foil was used for fiber deposition. The deposited fiber mats were dried for 72 h at room temperature and stored in a desiccant until the analysis.

### Characterization of nanofibers

#### Differential scanning calorimetry analysis

Differential scanning calorimetry analysis thermograms of drug, polymer, and nanofibers were obtained using a differential scanning calorimeter (Shimadzu DSC-60, Kyoto, Japan). The samples were heated from 25 to 200 °C at a rate of 10 °C/min under nitrogen atmosphere.

#### Encapsulation efficiency of electrospun nanofibers

Nanofibers (1 × 1 cm) were weighed and 1 mL of dichloromethane was added to dissolve fiber mats and 1 h later 7 mL of phosphate buffer solution (pH 7.4) was added to dissolve the drug released from the nanofibers (*n* = 3). After evaporation of DCM, the volume of each solution was completed to 10 mL with buffer and the amount of ampicillin trihydrate was analyzed using a UV spectrophotometer (Thermo Scientific Evolution 201). The encapsulation efficiency of the nanofibers was calculated using the following equation.

Encapsulation efficiency = (the amount of drug loaded/theoretical drug amount in the nanofiber) × 100.

#### In vitro drug release

The static method was used to evaluate the in vitro drug release of the nanofiber mats. Nanofiber mats (2 × 2 cm) were weighed and incubated in 5 mL of phosphate buffer solution (pH 7.4) at 37 °C (*n* = 3). All of the released medium was removed and 5 mL of fresh solution was added at predetermined time intervals. The amount of drug released was assayed using a UV spectrophotometer.

For calibration and validation of ampicillin trihydrate release, solutions were prepared at 2.5–30 µg/mL concentrations by making dilutions from a 200 µg/mL ampicillin trihydrate stock. Absorbance values were measured at a wavelength of 213 nm (*y* = 0.0309*x* + 0.0466, *r*^2^ = 0.9984).

#### Morphology of electrospun nanofibers

Nanofibers were firstly gold-coated and the morphology of the electrospun nanofibers was observed on a scanning electron microscope (QUANTA 400F Field Emission SEM, Holland). The average diameters of the resulting nanofibers were calculated by the measurement of 100 single nanofibers from SEM images using the ImageJ analysis software (National Institutes of Health, USA).

#### Mechanical properties

The mechanical properties, such as tensile strength and elongation at break values of the electrospun nanofibers (2 × 1 cm) were evaluated on a texture analyzer (TAXT Plus, Stable Micro Systems, United Kingdom) with an extension rate of 10 mm/s (*n* = 3). Tensile strength (mPa) and elongation at break (%) values of the nanofibers were calculated from the strain–stress curves.

#### Statistical analyses

All data were expressed as mean ± SD. Statistical analyses were performed using SPSS 20.0 for Windows (SPSS, Chicago, IL). The significance was evaluated with one-way ANOVA followed by Tukey’s post hoc test (SPSS 20.0). The data were considered significant at *p* < 0.05.
